# Hemispheric Coherence in ASD with and without Comorbid ADHD and Anxiety

**DOI:** 10.1155/2016/4267842

**Published:** 2016-04-04

**Authors:** A. Saunders, I. J. Kirk, K. E. Waldie

**Affiliations:** School of Psychology, The University of Auckland, Auckland 1010, New Zealand

## Abstract

There is a growing body of evidence suggesting that altered brain connectivity may be a defining feature of disorders such as autism spectrum disorder (ASD), anxiety, and ADHD. This study investigated whether resting state functional connectivity, measured by 128-channel EEG oscillation coherence, differs between developmental disorders. Analyses were conducted separately on groups with and without comorbid conditions. Analyses revealed increased coherence across central electrodes over the primary motor cortex and decreased coherence in the frontal lobe networks in those with ASD compared to neurotypical controls. There was increased coherence in occipital lobe networks in the ADHD group compared to other groups. Symptoms of generalised anxiety were positively correlated with both frontal-occipital intrahemispheric (alpha only) coherence and occipital interhemispheric coherence (alpha, approaching theta band). The patterns of coherence in the ASD pure group were different when comorbid conditions were included in the analyses, suggesting that aberrant coherence in the frontal and central areas of the brain is specifically associated with ASD. Our findings support the idea that comorbid conditions are additive, rather than being symptoms of the same disorder.

## 1. Introduction

Brain connectivity describes the pattern of links between regions of our brain. Connectivity is broadly split into functional connectivity (which describes the similarity of temporal characteristics of brain activity in different brain regions) or structural connectivity (the physical connections of regions) [1]. Communication and integration of segregated areas or networks of the brain are vital for the successful execution of cognitive and motor functions [2]. Measuring synchronisation or coherence of EEG while the brain is at rest is a common method with which to gauge cortical functional connectivity. Resting state connectivity measures allow investigators to study the flow of mental events in the absence of task performance (which requires the employment of task specific regions). Brain activation during resting state connectivity investigations can thus be used as a measure of baseline brain activity [3].

*Connectivity in ASD*. Autism spectrum disorder (ASD) is a lifelong neurodevelopmental condition that involves a spectrum of impairments in social, communication, and behavioural domains [4] that prevents the development of normal social interactions and relationships between the individual and others around them. Differential patterns of connectivity may be responsible for a mismatch of behaviour to environment in ASD. An underconnected system would be particularly disruptive to those higher-order psychological functions that require the coordination of many different types of information processing [5]. The difficulties that occur with an underconnected system would produce a multitude of problems with psychological functions (i.e., language, social skills, or executive functions) [6]. Together these problems may give rise to the difficulties evident in ASD.

*Neuroimaging Research on Connectivity in ASD*. There is a growing body of research showing aberrant connectivity in ASD. Altered patterns of connectivity are implicated in many brain regions [6]. Earlier studies using functional magnetic resonance imaging (fMRI) have revealed that those with ASD show functional underconnectivity between anterior and posterior regions [7]. Diffusor tensor imaging studies, which trace white matter tracts in the brain, have demonstrated altered structural connectivity in ASD. Compared to typically developing adults, those with ASD show increased mean and radial diffusion in white matter tracts forming corticocortical and interhemispheric connections [8]. This putatively indicates reduced fibre density of these tracts in those with ASD. However, other studies have failed to replicate these findings (e.g., [9, 10]). This discrepancy, in part, may be resolved by closely examining the characteristics of ASD within the spectrum. Recently, a study using fMRI demonstrated that connectivity may be related to the symptoms of ASD (which vary from individual to individual) rather than being a blanket feature of ASD itself. Specifically, those with more severe ASD symptoms show greater deviations in connectivity patterns from typically developing controls than those with less severe ASD symptoms [11]. Studies such as these highlight the importance of examining individual variation in disorders that are highly variable from individual to individual.

*Connectivity in Common Coexisting Conditions with ASD*. Altered connectivity is also thought to be present in obsessive compulsive disorder (OCD) and attention deficit/hyperactivity disorder (ADHD). In OCD, fMRI studies have demonstrated patterns of increased connectivity in the corticostriatal regions and frontosubcortical circuitry [12, 13]. In ADHD, there is a distinct pattern of underconnectivity between anterior and posterior regions [14] and reduced connectivity in the frontostriatal connections [15, 16]. In OCD and ADHD the patterns of altered connectivity seem to be associated with frontostriatal circuitry.

Taken together, resting state imaging studies have shown connectivity abnormalities in each disorder (ASD, ADHD, and OCD). Despite high comorbidity rates between the three disorders it is unclear how patterns of coherence present when two or more of these conditions present as a comorbid condition. The differential pattern of connectivity seen in these disorders suggests that specific brain regions may not be properly linked to each other.

*Connectivity and EEG.* Electroencephalography (EEG) is a useful tool in investigating connectivity using coherence measures. Coherence measures the amount of neural synchronisation between two electrodes (or groups of electrodes) on the scalp. High coherence between two EEG signals reflects cooccurrence of neuronal oscillations at the same frequency (suggesting functional integration between neural populations), whereas low coherence suggests independently active populations (suggesting functional segregation) [17]. Coherence can be determined from the power of oscillations in each frequency band (e.g., delta (1.5–3 Hz), theta (3.5–7.5 Hz), and alpha (8–12 Hz)). Power reflects the number of neurons that discharge in synchrony in each band [18]. The encoding of new information has been proposed to be reflected in theta oscillations, whereas alpha oscillations are associated with retrieval of long-term memories [18]. Power is also associated with task demands. As opposed to task-related research, the power of alpha increases and theta decreases in resting state studies [18].

Differences in coherence may measure default mode network activity, a system associated with rest. The default mode network is a specific group of brain areas that are active in the absence of task performance. A study by Greicius et al. [19] demonstrated that the posterior cingulate cortex and ventral anterior cingulate cortex show greater activity during resting states than during cognitive tasks. The authors theorised that this so-called default mode network is responsible for ongoing mental processes (such as working memory) during rest [19].

*Coherence in ADHD, Anxiety, and ASD*. Patterns of over- and underconnectivity are also evident in ASD using coherence measures, as measured in each power band. One study by Murias et al. [17] found elevated coherence (frontal and temporal regions) in the theta band and reduced coherence (frontal regions) in the lower alpha range (8–10 Hz). Another study found decreased interhemispheric delta and theta and decreased intrahemispheric delta and theta in the frontal regions [20].

Altered patterns of coherence are also a feature of ADHD. A study investigating coherence in ADHD found that at shorter interelectrode distances, children with ADHD had elevated intrahemispheric coherences in the theta band and reduced lateral differences in the theta and alpha bands. At longer interelectrode distances, ADHD children had lower intrahemispheric alpha coherences than controls. Frontally, ADHD children had interhemispheric coherences elevated in the delta and theta bands and reduced in the alpha band [21].

Coherence measures of OCD are in line with imaging studies demonstrating altered subcortical circuitry. To elaborate, there is a significant increase in the theta band in frontooccipital coherence in those with OCD versus typically developing controls [22]. In addition, there is decreased interhemispheric coherence in OCD [23]. However, the evidence for coherence anomalies in the broader anxiety category is unclear. There is some evidence to suggest that self-reported traits of anxiety are associated with decreased coherence using EEG [24]. Further research needs to be conducted to clarify patterns of connectivity in anxiety.

The current study investigated whether differences in resting state connectivity are shared by each condition or have different connectivity profiles from one another. We also sought to determine whether altered connectivity patterns are associated with specific behavioural characteristics. Given that altered coherence is associated with many neurobehavioural conditions, we expected to see a significant correlation between coherence values and scores on behavioural profiling measures.

## 2. Methods

### 2.1. Participants

The initial sample consisted of 47 subjects. One subject was excluded from the final analyses due to technical difficulties. All subjects had normal or corrected-to-normal vision and no history of head injury. The final sample (*n* = 46) consisted of 20 females and 26 males. Within each group the gender ratio was not evenly split (see Table 1). This is in line with the literature which demonstrates that autism is more common in males [25], and anxiety is much more common in females [26, 27]. The mean age of all participants was 25.48 years, with a range from 16.3 years to 46.5 years.

Subjects were recruited using advertisements placed around the University of Auckland City Campus and on a participant recruitment website. Subjects were also recruited through organisations such as Altogether Autism, the Phobic Trust, the Parent and Family Centre, and the ADHD Association. Once subjects contacted the researcher with their interest in the study, they were given an initial questionnaire to ensure they fit the study criteria. Participants were required to have no history of head injury and no history of comorbid depression or schizophrenia, be right-handed, and have English as their first language. Participants were given $20 in vouchers for their participation in this study.

Control subjects consisted of mainly undergraduate students who had no history of psychological illness (e.g., depression, anxiety). Subjects who fell into the ASD, anxiety, or ADHD groups were diagnosed with their respective conditions by a registered medical professional prior to participation (using diagnostic criteria such as the DSM). Diagnoses were confirmed at the testing session using the profiling tools described below. Subjects were also asked to disclose any coexisting conditions. From the 11 subjects in the ADHD group, 3 had an additional diagnosis of anxiety. In the ASD group, five subjects had a diagnosis of high functioning autism with no coexisting conditions; 6 had a diagnosis of high functioning autism and coexisting anxiety or OCD; and the remaining 2 had a diagnosis of coexisting high functioning autism and ADHD. The anxiety group included those with a diagnosis of anxiety or OCD. Seventeen participants were taking psychoactive medication at the time of participation (5: Concerta, 5: Ritalin, and 7: fluoxetine). Participants were asked not to take their medication on the day of participation in order to minimize the effect on EEG recording.

Participants were asked to answer six questionnaires that measured specific behaviours: (1) The Integrated Visual and Auditory Continuous Performance Task (CPT), which tests sustained attention to visual and auditory stimuli [28]; (2) The Behavioural Inhibition/Activation Scale (BIS/BAS), which identifies patterns of behaviour in an individual's personality [29] and consists of Behavioural Inhibition (BIS) or punishment sensitivity scale (e.g., “I worry about making mistakes”), reward responsiveness (e.g., “When good things happen to me, it affects me strongly”), drive (e.g., “I go out of the way to get things I want”), and fun seeking (e.g., “I am always willing to try something new if I think it might be fun”); (3) The Generalised Anxiety Disorder Scale (GADS) [30], which is a brief clinical measure for generalised anxiety; (4) The Obsessive Compulsive Inventory-Revised (OCI), an 18-item questionnaire that measures OCD symptoms [31]; (5) The Adult ADHD Self-Report Symptom Checklist (ASRS) [32], an 18-item self-report questionnaire which asks about clinical symptoms of ADHD as reported in the DSM-IV (e.g., “How often do you fidget or squirm when you have to sit down for long periods of time?”); and (6) The Autism Spectrum Quotient (ASQ) [33], a 50-item self-report questionnaire that measures autistic-like traits in the typically developing population.

### 2.2. Procedure

The experiment consisted of two trial blocks, eyes closed and eyes open. The order of blocks (i.e., eyes closed or eyes open first) was counterbalanced across participants. In the eyes open condition, participants were instructed to fixate on a black cross presented in the centre of a white screen. In the eyes closed condition participants were simply asked to close their eyes. Each block lasted for two minutes, giving a total EEG recording time of four minutes. Participants were instructed to remain still, try to relax, and not tense their muscles during the course of the experiment.

### 2.3. EEG Acquisition

EEG recordings were conducted in an electrically shielded room (Model L3000; Belling Lee, Enfield, England) using 128-channel Ag/AgCl electrode nets [34]. The Geodesic sensor net distributes electrodes from nasion to inion and from left to right mastoids at uniform intervals. EEG was recorded continuously (1000 Hz sample rate; 0.1–100 Hz analogue bandpass) with Electrical Geodesics Inc. amplifiers (300 MΩ input impedance). A Macintosh computer was used to acquire the data using NetStation software and this was then stored on the computer's hard disk. Electrode impedances were kept below 40 kΩ, an acceptable level for this system [34]. Common vertex (Cz) was used as a reference, resulting in a total of 129 electrodes.

### 2.4. EEG Processing

Coherence is a measure of the degree to which there is a cooccurrence of a particular frequency oscillation in the EEG recorded at different electrodes on the scalp. Coherence measures can give researchers an idea of the synchronisation of activation between locations [1].

The processing of the resting state data was conducted in two steps. The first step was to segment the data and separate into frequency bands. Data were segmented using in house software (WinView) to remove any artefacts according to the guidelines set out in [35]. Thereafter, the data was split into alpha (8–12 Hz) and theta (4–7 Hz) bands. The second step was to transform the data for each condition using a Fast Fourier Transform (ArcTanH(sqrt(y))) to calculate the variation of the correlation as a function of time in each power band. 512 ms windows, shifting across each block, were subject to a Fast Fourier Transformation to change the data from the time to the frequency domain. This resulted in four conditions for each participant: (1) alpha band: eyes closed; (2) alpha band: eyes open, (3) theta band: eyes closed; (4) theta band: eyes open.

### 2.5. Coherence Analysis

Pairs of electrodes were selected for coherence analysis (see Table 2 for a list of electrode pairs). Electrode pairs were selected using the international 10-20 system [36].

### 2.6. Statistical Analyses

All statistical analyses were conducted using SPSS version 20 software. The main analyses used were ANOVA and Pearson's correlations to investigate if there were any differences within or between subjects on coherence measures for each power band. Due to unequal sample sizes, Kruskall-Wallis nonparametric statistics were conducted on comorbid populations. All *p* values were deemed significant at the .05 level unless otherwise indicated. All significant effects were followed up by Bonferroni post hoc tests.

In order to increase statistical power, values for each pair of electrodes were averaged across area. For example, the frontal-frontal electrode pairs (F7-F8 and F3-F4) were averaged to produce one value for frontal-frontal coherence. Preliminary analyses revealed that there were no significant differences in coherence values between electrode pairs before they were averaged (*p* > .05).

## 3. Results

### 3.1. Alpha Eyes Closed

**Figure 1:**
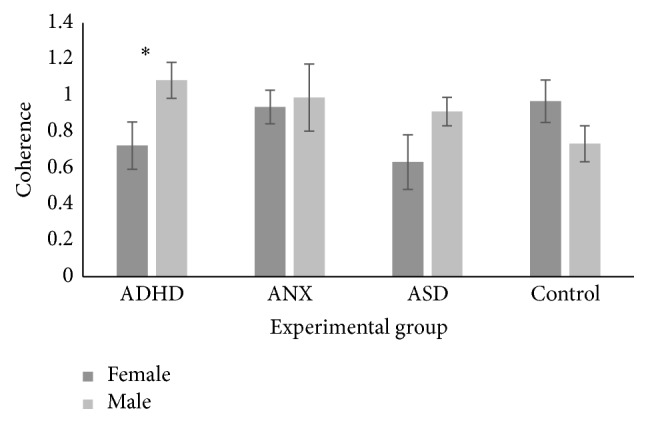
Interaction between gender and experimental group for the frontal-frontal interhemispheric coherence values in the alpha eyes closed condition. Significant effects at the .05 level are marked with an asterisk.

A 2 (gender) × 4 (ADHD, anxiety, ASD, and control) multivariate ANOVA was conducted on the eyes closed alpha band coherence values. There were no significant effects for any coherence values for either gender or experimental group. There was an interaction between gender and experimental group for the frontal-frontal interhemispheric coherence scores that was approaching significance, *F*(3,39) = 2.80, *p* = .053. For the ADHD group only, males (M = 1.08, SE = .10) had significantly higher coherence values than females (M = .72, SE = .13, *p* = .035), as shown in Figure 1.

### 3.2. Alpha Eyes Open

A 2 (gender) × 4 (ADHD, anxiety, ASD, and control) multivariate ANOVA was conducted on the eyes open alpha band coherence values. There was a significant main effect of gender for the frontal-frontal intrahemispheric coherence values, *F*(1,38) = 7.81, *p* = .008, where females (M = 1.20, SD = .37) had lower coherence than males (M = 1.44, SD = .43). There was also an interaction between experimental group and gender that was approaching significance for central-central interhemispheric coherence scores, *F*(3,38) = 2.52, *p* = .072. For males only, those with ADHD (M = .77, SD = .13) had higher coherence values than controls (M = .24, SD = .13, *p* = .044). In addition, for those with ADHD only, females (M = .25, SE = .17) had lower coherence values than males (*p* = .015).

### 3.3. Theta Eyes Closed

A 2 (gender) × 4 (ADHD, anxiety, ASD, and control) multivariate ANOVA was conducted on the eyes closed theta band coherence values. There was a significant main effect of gender for central-central interhemispheric coherence scores, *F*(1, 39) = 5.44, *p* = .025, where females (M = .51, SE = .06) had higher coherence than males (M = .32, SE = .06). There was also a significant main effect of experimental group for central-central interhemispheric coherence values, *F*(3,39) = 3.32, *p* = .030. Post hoc tests, however, reveal no differences between the groups.

### 3.4. Theta Eyes Open

A 2 (gender) × 4 (ADHD, anxiety, ASD, and control) multivariate ANOVA was conducted on the eyes open theta band coherence values. There was a significant main effect of gender for central-central interhemispheric coherence values, *F*(1,39) = 5.28, *p* = .027. Females (M = .56, SE = .07) had higher coherence values than males (M = .33, SE = .07). There was a main effect of experimental group for the frontal-frontal intrahemispheric coherence values that was approaching significance, *F*(3,39) = 2.39, *p* = .083. There was also a main effect of experimental group for occipital-occipital interhemispheric coherence that was approaching significance *F*(3,39) = 2.73, *p* = .057. Those with ASD (M = 1.41, SE = .47) had lower coherence than those with ADHD (M = 1.89, SE = .40, *p* = .076).

There was an interaction between gender and experimental group for central-central interhemispheric coherence values that was approaching significance, *F*(3,39) = 2.49, *p* = .074. For those with ASD only, males (M = .33, SE = .09) had lower coherence scores than females (M = .87, SE = .17, *p* = .010).

### 3.5. Nonparametric Analyses

**Figure 2:**
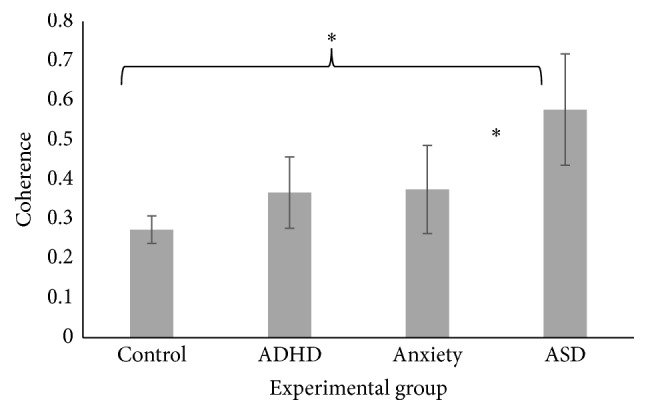
Significant main effect of pure experimental groups in the alpha central-central interhemispheric eyes closed condition. Significant effects at the .05 level are marked with an asterisk.

A Kruskall-Wallis test was conducted to investigate whether coherence values were different between pure experimental groups. The results showed a significant difference between groups for the alpha central-central interhemispheric eyes closed condition, *χ* ^2^(3) = 7.83, *p* = .050. The ASD group (M = .58, SE = .32) had higher coherence than the control group (M = .27, SE = .03) for the alpha central-central interhemispheric eyes closed condition, *U* = 8.00, *p* = .020, and the anxiety (M = .37, SE = .11) group, *U* = 7.00, *p* = .027, as shown in Figure 2.

The theta frontal-frontal intrahemispheric coherence eyes closed values were approaching significance, *χ* ^2^(3) = 6.93, *p* = .074. The ASD group (M = .82, SE = .12) had lower coherence scores than the control group (M = 1.19, SE = .07, *U* = 8.00, *p* = .020) and the anxiety group (M = 1.21, SE = .08, *U* = 6.00, *p* = .020).

### 3.6. Correlations

Pearson's correlations were conducted between the following variables and coherence for the alpha and theta bands: Continuous Performance Task (CPT); Behavioural Inhibition Scale; response reward; drive; fun seeking; GAD; Obsessive Compulsive Inventory (OCI); Adult Self-Report ADHD scale (ASRS); and the ASQ (see Tables 3 and 4).

There was a significant moderate positive correlation between frontal-occipital intrahemispheric eyes closed and the GAD, *r* = .30, *p* = .041, a moderate positive relationship between the frontal-occipital intrahemispheric eyes open and fun seeking scores, *r* = .30, *p* = .038, a moderate negative correlation between the central-parietal intrahemispheric eyes open and the CPT, *r* = −.39, *p* = .006, a moderate positive correlation between the frontal-frontal interhemispheric eyes open and fun seeking scores, *r* = .34, *p* = .023, and the ASRS, *r* = .37, *p* = .023, a moderate negative correlation between the central-central interhemispheric eyes open and the CPT, *r* = −.42, *p* = .004, and fun seeking scores *r* = .29, *p* = .045, a moderate positive correlation between the parietal-parietal interhemispheric eyes open and the ASRS, *r* = .32, *p* = .027, and finally a moderate positive correlation between the occipital-occipital interhemispheric eyes open and the GAD, *r* = .39, *p* = .006, and the ASRS, *r* = .38, *p* = .009.

There was a significant moderate negative correlation between the frontal-occipital intrahemispheric eyes open and CPT scores, *r* = −.37, *p* = .021, and the central-parietal intrahemispheric eyes open condition and CPT scores, *r* = −.44, *p* = .002, a moderate positive correlation between the parietal-parietal interhemispheric eyes closed and BIS scores, *r* = .32, *p* = .032, and the GAD scores, *r* = .44, *p* = .026, and a weak negative correlation between the occipital-occipital interhemispheric eyes open and ASQ scores, *r* = −.29, *p* = .048.

## 4. Discussion

The main aim of this study was to investigate whether resting state functional connectivity, measured by 128-channel EEG oscillation coherence, differs between developmental disorders. Analyses were conducted separately on groups with and without comorbid conditions. Overall, the analyses revealed differential patterns of coherence in the ASD group (in particular, higher coherence in the primary motor cortex, and lower coherence in the frontal lobe networks compared to neurotypical controls). In addition, there was increased coherence in the occipital lobe in the ADHD group. These patterns of altered connectivity were expected given the research outlined above. Each will now be discussed in turn.

### 4.1. Pure ASD, ADHD, Anxiety, and Neurotypical Controls

Differences in resting state coherence were apparent when the groups included only the pure form of each disorder (i.e., removing those with comorbid conditions from the analyses). Those with ASD have higher coherence in alpha across central interhemispheric sites when the eyes were closed, relative to those with only anxiety or controls. That the difference is apparent in the eyes closed condition is consistent with previous research that demonstrates that alpha power increases when subjects close their eyes [37]. The central interhemispheric electrodes (C3 and C4 using the international 10-12 system) are located over the primary somatosensory cortex, in the parietal lobe, which is responsible for processing touch and sensation in addition to keeping track of the location of your body parts [38]. As mentioned previously, higher coherence reflects a higher degree of synchrony of neural oscillations in a particular band (the amount of neural activity dedicated to that process) [18]. Higher alpha coherence in this region is interesting given that those with ASD have well-documented sensory hypersensitivity, especially to touch [39], and have aberrant body movements [4]. Therefore, the results of the current study suggest that the behavioural profile of sensory hypersensitivity to stimuli may be reflected in increased alpha power across the primary somatosensory cortex.

The theta frontal intrahemispheric coherence values when the eyes were closed were lower in the ASD group versus those with anxiety or controls. This finding is consistent with research demonstrating high theta power in neurotypical controls during rest or relaxation [18]. To illustrate, one study found significantly higher theta power in the frontal and temporal-central areas of the brain during meditation versus rest [40], suggesting that theta increases as higher relaxation occurs. This pattern of activity was not evident in the ASD group. The electrodes used in the current study for the theta frontal intrahemispheric coherence analysis were all located over the frontal lobe. The frontal lobe is associated with executive function, complex tasks that require working memory, motor planning, and logical and emotional attention [41]. A pattern of hypocoherence in the Fp1-F3 and Fp2-F4 electrodes has been specifically associated with less efficient integration of motor actions and logical attention [42]. Those with ASD have well-documented difficulties with motor actions and logical attention, especially joint attention [43, 44]. Thus, these behavioural difficulties could be reflected in lower overall theta power in the frontal lobe.

### 4.2. Group Level Analyses including Comorbid Conditions

There were few differences in resting state connectivity between groups when comorbid conditions were included. One effect that was trending toward significance was that the ADHD group had higher interhemispheric theta (eyes open) coherence across the occipital lobe relative to the ASD group. While a pattern of increased connectivity suggests more dedicated neural processing, a pattern of coherence higher than that seen in neurotypical controls suggests that the processes are less flexible in these individuals [45]. This finding is consistent with previous literature demonstrating increased theta coherence in the interhemispheric occipital regions in ADHD [46]. The occipital lobe is primarily involved in visual processing. As such, a pattern of hypercoherence in this area has been shown to result in a lack of flexibility in visual sensations [42]. This finding fits with the behavioural profile of those with ADHD who experience difficulties with visual attention [47]. The results of the current study suggest that visual attention difficulties in ADHD may be driven by hypocoherence in the occipital lobe.

This interhemispheric increase in coherence may also be associated with the established patterns of atypical laterality in ADHD. While typically developing controls show hemispheric specialisation (i.e., higher involvement of one hemisphere over the other) for tasks such as language, this hemispheric specialisation is not as pronounced in ADHD (e.g., [48, 49]). It is possible that the pattern of hypocoherence seen in the occipital lobe in those with ADHD may be, in part, caused by atypical laterality in the ADHD brain.

In the alpha band with eyes open, females had lower intrahemispheric coherence than males in the frontal regions. In the theta band with eyes closed and eyes open, females had higher interhemispheric coherence scores than males in the central region. Gender differences in EEG coherence have been reported in the past and may be due to anatomical differences in brain structure and development between the sexes [50]. As these differences are limited to the central and frontal regions, both regions where aberrant connectivity was found in those with ASD, it is also possible that the gender imbalance in the ASD group contributed to this effect.

An overall aim of the current study is to determine whether comorbid conditions in ASD are separate (i.e., additive) or simply symptoms of the ASD itself (i.e., a misdiagnosis). Some theorists and practitioners believe that hyperactivity or anxiety when presented together with ASD is simply a characteristic of the ASD [51]. If this were the case, then we would expect patterns of coherence differences evident in the ASD only group to be either very similar or more pronounced when additional conditions are added to the analyses. Evidence from the current data suggests that this is not the case. The patterns of coherence in the ASD pure group are no longer present when additional conditions are included in the analyses. Data from the pure group analyses show that coherence differences are limited to the sensory motor cortex and frontal cortex in ASD and the occipital lobe in ADHD. Therefore, the patterns of aberrant coherence in the frontal and central areas of the brain are specifically associated with ASD and a different pattern of coherence is produced when ASD presents as a comorbid condition. This suggests that the two conditions are additive.

### 4.3. Correlational Analyses

The final question of interest was whether coherence values were associated with the phenotype of the various disorders (i.e., hyperactivity, anxiety, and social skill difficulties). Differences in coherence scores are indeed associated with characteristics of ADHD and generalised anxiety. Overall, there was a pattern of increased coherence associated with increased characteristics of ADHD and anxiety. In ASD, however, while there was less of an association, patterns of decreased coherence were somewhat associated with an increase in symptoms. Below the CPT, ASRS, fun seeking, and GAD coherence results will be discussed.

The regions associated with increased coherence on the CPT and fun seeking scales were similar. As scores on the Continuous Performance Task (which measures visual and auditory attention) decreased (demonstrating more severe symptoms of ADHD), coherence scores increased in the following areas: the central-parietal intrahemispheric (in both alpha and theta) and the central interhemispheric (alpha, with theta trending toward significance) regions; and the frontal interhemispheric (trending toward significance in alpha, theta) and the frontal-occipital intrahemispheric (theta only) regions. As determined in Section 3, fun seeking scores were a significant predictor of ADHD. Using this measure we were able to distinguish those with ADHD from neurotypical controls, where those with ADHD had higher fun seeking scores. As scores on the fun seeking scale increased, so did coherence for the frontal-occipital intrahemispheric (alpha, theta trending toward significance), frontal interhemispheric (alpha), and the central interhemispheric (alpha) regions.

As scores on the ASRS increased, coherence in the frontal interhemispheric (alpha, theta trending), parietal interhemispheric (alpha only), and the occipital interhemispheric (alpha only) regions increased. There was a trend towards an effect in the frontal intrahemispheric and the central intrahemispheric areas (with both increasing simultaneously in alpha only). This finding is in line with the group level analyses discussed earlier that demonstrated increased coherence in the occipital area for those with ADHD.

Increased interhemispheric coherence in the frontal and central areas has been shown to be associated with lack of flexibility in logical attention. Increased coherence in the central-parietal region has been associated with less flexible sensorimotor integration, and, finally, increased coherence in frontal-occipital coherence has been associated with less flexible integration of visual sensations [42]. As mentioned previously, a pattern of coherence higher than that seen in neurotypical controls suggests that the processes are less flexible in these individuals [45]. Accordingly, behavioural studies have established a pattern of difficulties with logical attention [52], sensorimotor integration (in particular, motor preparation, timing and adjustment, and delayed motor action processes), and visual sensations [47, 53] in ADHD.

Increases in coherence scores are also associated with characteristics of anxiety. As scores on the GAD increased, so did coherence scores in the frontal-occipital intrahemispheric (alpha only) region and the occipital interhemispheric region (alpha, approaching theta band). In the theta band as scores on the GAD increased, so did coherence values in the parietal interhemispheric region. As scores on the OCI increased, there was a trend toward an increase in parietal interhemispheric coherence scores in the alpha band and central interhemispheric scores in the theta band. Increased coherence in these regions has been linked to a lack of flexibility in visual sensations [42].

While there is little literature to suggest a link between less flexible visual sensations and anxiety, it is well known that those with anxiety have an automatic and instinctive reaction to objects or situations that may or may not represent true danger [54]. To illustrate, one study presented participants with anxiety with either positive or negative motivational (i.e., a “win” or a “loss” in a game) stimuli. Those with anxiety were significantly faster to respond to the negative versus positive stimuli, which suggests earlier processing for negative emotional states [55]. In addition, those with anxiety show a high vigilance toward threatening stimuli (where threatening stimuli are processed much more quickly than nonthreatening stimuli) [56]. This pattern of early processing for negative over positive stimuli could be driven by less flexible response to visual stimuli, where there is automaticity for processing negative or threat-based stimuli which is driven by a pattern of hypercoherence in underlying brain networks. To the best of our knowledge, there are no studies investigating resting state EEG coherence in anxiety. Thus, this is the first study to demonstrate that increased coherence in the frontal-occipital, occipital, and parietal regions is associated with increased behaviour of anxiety.

There is less of an association between scores on the ASQ and coherence values. As scores on the ASQ increased, coherence values decreased in the occipital interhemispheric region, and there was a trend toward significance in the theta band for the frontal intrahemispheric region (where coherence scores decreased as ASQ scores increased). Decreased theta coherence in the frontal region fits with the analyses conducted on ASD pure groups demonstrating the same effect. However, the decreased coherence in the occipital lobe was not found in the initial analyses. This may be due to small sample sizes contributing to lower statistical power. Interestingly, this pattern of decreased coherence in the occipital lobe is the exact opposite to the profile seen in those with ADHD (where coherence was increased). Therefore, while a lack of flexibility to visual sensations is seen in ADHD, decreased coherence in ASD suggests dysfunctional integration of information related to visual sensations.

Coherence measures underlying connectivity of brain networks, that is, the amount of neural activity communicated between regions [17]. The current study found overall patterns of decreased coherence, therefore suggesting decreased connectivity in those with ASD. While an initial overgrowth in brain volume is evident in ASD in early childhood, this overgrowth slows and brain volumes are comparable to healthy controls in adulthood [57]. This suggests that the neural pruning mechanisms in the ASD brain do not function as they do in neurotypical adults, which may result in patterns of decreased connectivity in specific pathways. This finding is in line with an established body of literature demonstrating decreased connectivity in ASD [7, 58]. Interestingly, connectivity is decreased in the areas of the brain which reflect emotional and sensorimotor processing, which processes those with ASD display deficits. Decreased neural integration for these functions may underlie these difficulties.

### 4.4. Practical Implications

It is possible to alter the strength of neural coupling (i.e., connectivity) in the brain with training. The brain is plastic and therefore responds to change with physical alterations of function or anatomy [59]. For example, research has demonstrated that training on a working memory task increased white matter tracts in the parietal region and the corpus callosum in the brain [60]. Another study found that relational reasoning training increased frontoparietal and parietal-striatal connectivity at rest [61]. It is possible that intensive targeted training may help to increase connectivity in those with ASD, resulting in an improvement in behaviour. However, those with ADHD and anxiety show overall patterns of increased connectivity. It is unclear whether training would help to decrease the connectivity in this instance. Further research is needed to establish whether training would improve patterns of hyperconnectivity in those affected.

### 4.5. Limitations

There are limitations in the current study that warrant mention. The first limitation is our small sample size. A larger-scale study investigating the pure groups to their comorbid counterparts would provide valuable information. Secondly, the individuals in the current study were high functioning. All individuals had a normal IQ with no language deficits. It could be argued that individuals on the ASD spectrum who are higher functioning show a completely different connectivity profile compared to their lower functioning counterparts. Thus, the results of the current study may not be applicable to the entire ASD population, but rather a specific, higher functioning subset who once met the old diagnostic criteria of “Asperger's syndrome.” In addition, some participants were taking psychoactive medication at the time of participation. While participants were asked not to take their medication on the day of participation to minimize the impact on EEG recording, it is possible that participants performance was still affected.

## 5. Conclusions

Overall, the results of the current study demonstrate patterns of decreased coherence in those with ASD and patterns of increased coherence in ADHD and anxiety. Therefore, in their pure form, those with ASD, ADHD, anxiety, and neurotypical controls have different resting state functional connectivity profiles. These patterns are no longer evident when comorbid conditions are included in the analyses, which suggest that additional conditions cannot be accounted for through the ASD diagnosis. In ASD, reductions in functional connectivity potentially underlie a lack of neural integration for tasks relating to sensorimotor and emotional processing. In ADHD and anxiety, patterns of increased coherence potentially reflect a lack of flexibility in the processing of visual stimuli, attention, and sensorimotor integration. For those with anxiety, the increase in connectivity is limited to visual sensations, which is likely related to their increased sensitivity to threat stimuli.

## Figures and Tables

**Table 1:** Mean age and IQ with standard deviations in parentheses and gender distribution for each experimental group in the resting state task.

	ADHD		Anxiety		ASD		Control	
	Mean	SD	Mean	SD	Mean	SD	Mean	SD
Age	23.36	(3.93)	25.82	(8.50)	25.88	(8.78)	26.68	(7.62)
IQ	116.60	(8.46)	116.56	(8.62)	108.64	(13.18)	108.50	(13.52)
Female	4		8		3		5	
Male	7		2		10		7	

**Table 2:** All electrode pairs selected for analysis, the area of the brain they are located over, and whether they are interhemispheric or intrahemispheric connections.

Electrode pair	Area	Hemisphere
F3-O1	Frontal-occipital	Intrahemispheric
F4-O2	Frontal-occipital	Intrahemispheric
Fp1-F3	Frontal polar-frontal	Intrahemispheric
Fp2-F4	Frontal polar-frontal	Intrahemispheric
C3-P3	Central^*∗*^-parietal	Intrahemispheric
C4-P4	Central^*∗*^-parietal	Intrahemispheric
Fp1-Fp2	Frontal polar-frontal polar	Interhemispheric
F7-F8	Frontal-frontal	Interhemispheric
F3-F4	Frontal-frontal	Interhemispheric
C3-C4	Central^*∗*^-central^*∗*^	Interhemispheric
P3-P4	Parietal-parietal	Interhemispheric
O1-O2	Occipital-occipital	Interhemispheric

^*∗*^Please note that there exists no central lobe and this is simply a reference to the placement of the electrodes.

**Table 3:** Correlations between coherence measures for each area, with eyes open or closed for the alpha band.

Alpha area	Continuous Performance Task	Behavioural Inhibition Scale	Response reward	Drive	Fun seeking	GAD	Obsessive Compulsive Inventory	Adult Self-Report ADHD scale	Autism Spectrum Quotient
Frontal-occipital intrahemispheric									
Eyes closed	0.008	0.157	0.041	−0.02	−0.226	.**299** ^*∗*^	0.162	0.095	0.168
Eyes open	−0.214	−0.062	−0.063	−0.215	.**303** ^*∗*^	−0.054	−0.055	0.137	−0.072
Frontal-frontal intrahemispheric									
Eyes closed	0.049	−0.059	−0.001	−0.052	0.037	−0.008	−0.04	0.118	−0.205
Eyes open	−0.086	−0.031	0.116	−0.091	0.157	0.002	−0.032	**0.265** ^*∗∗∗*^	−0.246
Central-parietal intrahemispheric									
Eyes closed	−0.172	0.056	0.039	−0.062	−0.015	0.035	−0.014	0.011	0.16
Eyes open	**−.393** ^*∗∗*^	−0.026	0.151	0.051	0.225	−0.187	−0.139	0.083	0.041
Frontal-frontal interhemispheric									
Eyes closed	−0.143	−0.04	−0.119	−0.23	0.062	0.082	−0.05	0.232	−0.083
Eyes open	**−0.246** ^*∗∗∗*^	−0.057	0.062	−0.079	**.335** ^*∗*^	−0.036	0.016	.**336** ^*∗*^	−0.201
Central-central interhemispheric									
Eyes closed	−0.089	0.193	0.054	0.151	0.085	0.016	−0.183	0.073	0.028
Eyes open	**−.417** ^*∗∗*^	−0.021	0.143	−0.215	**.294** ^*∗*^	−0.167	−0.225	**0.251** ^*∗∗∗*^	−0.019
Parietal-parietal interhemispheric									
Eyes closed	−0.127	0.043	0.077	0.079	0.092	0.201	0.086	0.206	0.084
Eyes open	−0.103	0.232	0.073	−0.058	0.187	0.277	**0.263** ^*∗∗∗*^	**.322** ^*∗*^	0.168
Occipital-occipital interhemispheric									
Eyes closed	−0.134	−0.08	0.118	−0.04	0.054	0.113	0.009	0.197	−0.07
Eyes open	0.001	0.048	0.158	0.036	0.129	.**392** ^*∗∗*^	0.202	**.378** ^*∗∗*^	−0.101

^*∗*^Significant at the .05 level. ^*∗∗*^Significant at the .001 level. ^*∗∗∗*^Approaching significance at the <.1 level.

**Table 4:** Correlations between coherence measures for each area, with eyes open or closed for the theta band.

Theta area	Continuous Performance Task	Behavioural Inhibition Scale	Response reward	Drive	Fun seeking	GAD	Obsessive Compulsive Inventory	Adult Self-Report ADHD scale	Autism Spectrum Quotient
Frontal-occipital intrahemispheric									
Eyes closed	0.051	0.104	−0.047	0.058	−0.136	0.065	−0.034	−**0.253** ^*∗∗∗*^	−0.056
Eyes open	**−.336** ^*∗*^	−0.122	−0.013	−0.043	**0.257** ^*∗∗∗*^	−0.145	−0.075	0.167	0.059
Frontal-frontal intrahemispheric									
Eyes closed	0.153	−0.014	0.013	−0.019	−0.103	0.126	0.109	0.005	−0.236
Eyes open	0.019	−0.022	0.136	−0.119	0.017	0.13	0.054	0.187	**−0.254** ^*∗∗∗*^
Central-parietal intrahemispheric									
Eyes closed	−0.122	0.107	0.23	0.18	−0.087	0.11	−0.024	−0.057	0.121
Eyes open	−.**439** ^*∗∗*^	0.076	**0.278** ^*∗∗∗*^	**0.265** ^*∗∗∗*^	0.056	0.06	−0.035	0.2	0.056
Frontal-frontal interhemispheric									
Eyes closed	−0.004	0.093	**−0.26** ^*∗∗∗*^	−0.11	−0.208	0.14	0.044	0.045	0.036
Eyes open	−0.105	0.032	−0.013	−0.147	0.121	0.038	0.016	0.053	−0.083
Central-central interhemispheric									
Eyes closed	0.014	**0.267** ^*∗∗∗*^	−0.01	0.098	−0.209	0.174	−0.075	−0.032	0.186
Eyes open	**−0.259** ^*∗∗∗*^	0.037	−0.1	−0.073	0.096	−0.065	**−0.265** ^*∗∗∗*^	0.006	0.029
Parietal-parietal interhemispheric									
Eyes closed	−0.027	**.324** ^*∗*^	0.094	0.141	−0.113	.**440** ^*∗∗*^	0.227	0.162	0.192
Eyes open	−0.086	0	−0.002	−0.103	0.208	0.153	0.048	0.152	−0.047
Occipital-occipital interhemispheric									
Eyes closed	−0.048	0.022	0.14	−0.022	−0.091	**0.266** ^*∗∗∗*^	0.024	0.156	−0.163
Eyes open	0.033	−0.148	0.066	0.08	0.161	0.138	−0.139	0.231	**−.290** ^*∗*^

^*∗*^Significant at the .05 level. ^*∗∗*^Significant at the .001 level. ^*∗∗∗*^Approaching significance at the < .1 level.
